# Clinical, pathological, and radiological features of 80 pediatric diffuse intrinsic pontine gliomas: A single-institute study

**DOI:** 10.3389/fonc.2023.1007393

**Published:** 2023-02-07

**Authors:** Peng Zhang, Yunyun Duan, Guocan Gu, Liying Qu, Dan Xiao, Tianshu Xi, Changcun Pan, Ya’ou Liu, Liwei Zhang

**Affiliations:** ^1^ Department of Neurosurgery, Beijing Tiantan Hospital, Capital Medical University, Beijing, China; ^2^ China National Clinical Research Center for Neurological Diseases, Beijing, China; ^3^ Beijing Neurosurgical Institute, Beijing, China; ^4^ Department of Radiology, Beijing Tiantan Hospital, Capital Medical University, Beijing, China

**Keywords:** diffuse intrinsic pontine glioma, H3K27 alteration, overall survival, MRS, radiomics

## Abstract

**Objective:**

Diffuse intrinsic pontine gliomas (DIPGs) are rare but devastating diseases. This retrospective cross-sectional study aimed to investigate the clinical, radiological, and pathological features of DIPGs.

**Materials and methods:**

The clinical data of 80 pediatric DIPGs under clinical treatment in Beijing Tiantan Hospital from July 2013 to July 2019 were retrospectively collected and studied. A follow-up evaluation was performed.

**Results:**

This study included 48 men and 32 women. The most common symptoms were cranial nerve palsy (50.0%, 40/80 patients) and limb weakness (41.2%, 33/80 patients). Among the 80 patients, 24 cases were clinically diagnosed, 56 cases were pathologically verified, and 45 cases were tested for H3K27 alteration status, with 34 H3K27 alteration cases confirmed. Radiological results indicated that enhancement was common (65.0%, 52/80 patients). Cho/Cr was of predictive value for H3K27 alteration status (*P* = 0.012, cutoff value = 2.38, AUC = 0.801). Open cranial surgery followed by further chemotherapy and radiotherapy was beneficial for patients’ overall survival. Cox regression analysis indicated H3K27 alteration to be the independent prognostic influencing factor for DIPGs in this series (*P* = 0.002).

**Conclusion:**

DIPGs displayed a wide spectrum of clinical and imaging features. Surgery-suitable patients could benefit from postoperative comprehensive therapy for a better overall survival. H3K27 alteration was the independent prognostic influencing factor for DIPGs.

## Introduction

Brainstem gliomas (BSGs) are defined as a heterogeneous group of gliomas that arise from the midbrain, pons, or medulla. BSGs show a bimodal peak of age distribution, with the first peak in the latter half of the first decade and the second peak in the fourth decade (1). BSGs account for approximately 20% of central nervous system tumors in children, and 80% of them are diffuse intrinsic pontine gliomas (DIPGs) (2).

The prognosis for diffuse BSGs has been very poor, compared with a better prognosis in focal low-grade gliomas of the midbrain or focal gliomas in the dorsal bulbo-medullary or pons. Pediatric DIPGs, with a median overall survival of 9–12 months, have been a main research focus for the past 50 years due to their inoperability and resistance to chemotherapy and radiotherapy (3). Whether chemotherapy and radiotherapy could improve prognosis is still controversial. Approximately 80% of pediatric DIPGs harbor H3K27 alterations. Brain tumors that harbor H3K27 alterations seem to have a particularly poor prognosis. In order to classify this new biological feature of brain tumors, a new subtype “diffuse midline glioma, H3K27 altered (DMG-H3K27-alt)” was termed (4). However, the radiomics features of DIPGs, as a pretreatment non-invasive measurement, especially for the DMG-H3K27-alt types, are less known.

DIPGs are a wide range of diseases defined by MR imaging. Histopathology with molecular features is the gold standard for diagnosis. A new study indicated the histone alteration status of predictive value for prognosis (3). Due to the risks of biopsy or open cranial surgery for DIPGs, MRI has been used for the diagnosis of DIPGs for decades. Whether MR features are correlated with histone alteration status and prognosis in DIPGs is still unknown. Furthermore, whether DIPG patients could benefit from chemotherapy and radiotherapy still needs more exploration. In order to obtain a better understanding of the clinical and imaging features of DIPGs, we conducted this retrospective cross-sectional study.

## Materials and methods

This study was approved by the Scientific Review Board of the IRB in our hospital. All DIPGs with intact clinical data and MR imaging data were included from July 2013 to July 2019. Clinical information, including age, sex, symptom duration before diagnosis, neurological findings, pathological findings, natural history, treatment with chemotherapy, and treatment with radiotherapy, was retrospectively collected. Patients were diagnosed with DIPG according to MRI images, clinical manifestations, and intact clinical records. Patients without magnetic resonance spectrum results or without intact clinical data were excluded. All the histopathological results and molecular pathological results were reviewed by an experienced team of neuropathologists at Beijing Tiantan Hospital, Capital Medical University.

### Imaging review

Imaging evaluation was independently performed by two senior radiologists and one neurosurgeon. A consensus opinion was utilized if there were discrepancies among the reviewers; all images were reviewed again and an agreement among the reviewers was reached. Imaging information included maximum tumor size in the cross section, percentage of pons involved in the cross section, tumor margin, eccentric position, T2 hypointensity, heterogeneity, diffuse restriction, hemorrhage, peritumoral edema, necrosis, cystic, enhancement characteristics, spectroscopy features, hydrocephalus, supratentorial peri-ventricular edema, presence of stripes in non-necrotic T2 hyperintensity regions in the cross section of the pons, location of medulla involvement, and location of midbrain involvement. Diffuse intrinsic pontine glioma was defined as having its epicenter in the pons and typically involving more than 50% of its surface according to the imaging features in MRI. Only diffuse intrinsic pontine gliomas were included in this study for further analysis. Quantitative analysis of diffusion, spectroscopy data, and correlation with other variables was performed.

### Histone alteration status

Cases were pathologically verified through stereotactic biopsy or open cranial biopsy. In addition to histopathological diagnosis, immunohistochemistry (IHC) assessment of histone alteration status was also performed. The IHC method utilizes a polyclonal, mutant-specific antibody that recognizes the product of all H3K27 variants, and a positive result was visualized microscopically as strong nuclear staining of the tumor cells (5).

### Statistical analysis

Univariable and multivariable analyses of imaging features (quantitative value of diffuse restriction and spectroscopy data, external pontine tumor location, and T2 pontine stripe features), clinical data, and histone status relative to overall survival (OS, diagnosis until death) were performed using Cox proportional hazards regression. The hazard ratio (HR) and odds ratio (OR) for OS and histone status were summarized. Variables with *P <*0.05 in the univariable analysis were chosen for multivariable analysis when applicable. Correlation analysis between histone status and other variables was also performed. Correlations with a value of *P* < 0.05 were considered to be of statistical significance. Paired *t*-tests or independent sample *t*-tests were used for quantitative data; *P <*0.05 was considered to be of statistical significance. The chi-square test was used for nominal data, and *P <*0.05 was considered to be of statistical significance.

## Results

### Demographics, clinical, and imaging features

#### Demographics and overall survival

A total of 80 patients who met the criteria were included, consisting of 48 men and 32 women. The median age was 7 years old (range between 2 and 14 years). The demographic data and correlation with OS are summarized in **Table 1**.

**Table 1:** Clinical parameters correlated with OS of 80 DIPGs.

Characteristics		No. (%) of patients	*P-*value
Sex			0.684
	Male	48 (60.0)	
	Female	32 (40.0)	
Symptom duration time before diagnosis			0.048
	≤1 month	29 (51.8)	
	>1 month	27 (48.2)	
Neurological findings			
	Cranial nerve palsy	40 (50.0)	0.731
	Limb weakness	33 (41.2)	0.839
	Limb sensory disturbance	10 (12.5)	0.249
	Ataxia	26 (32.5)	0.452
Treatment			0.00*
	Clinical observation	24	
	Open cranial cytoreductive surgery or stereotactic biopsy	18	
	Stereotactic biopsy plus radiotherapy and/or chemotherapy	6	
	Open cranial cytoreductive surgery plus radiotherapy and/or chemotherapy	32	

*Indicates P <0.01.

#### Clinical data and overall survival

Prediagnostic symptom duration time ranged from 1 week to 3 years (with a median duration time of 1 month, **Table 1**). The most common first symptom before diagnosis was lower cranial nerve palsy, followed by limb weakness and ataxia. A shorter prediagnostic symptom duration time indicated a much shorter overall survival (*P* = 0.048).

Among the 80 patients, 56 cases were pathologically verified through stereotactic biopsy or open cranial biopsy. Pathology results were reviewed back-to-back by two senior pathologists to reach an agreement. Totally, 56 cases were verified with pathology and a total of 45 cases were tested for H3K27 alteration status with 31 alteration-positive cases. The correlation between overall survival and pathological results is listed in **Table 2**. Higher tumor grade was correlated with the presence of necrosis on MRI imaging (*P* = 0.024) and indicated a much shorter overall survival time (*P* = 0.043).

**Table 2:** Pathological parameters correlated with OS of 56 DIPGs.

Characteristics		No. (%) of patients	*P-*value
Pathological findings			0.043*
	Astrocytoma (WHO grade II)	8 (14.3)	
	Oligodendroastrocytoma(WHO grade II)	5 (8.9)	
	Anaplastic oligodendroastrocytoma (WHO grade III)	12 (21.4)	
	Diffuse midline glioma with H3K27 alteration (WHO grade IV)	31 (68.9)	
H3K27M			0.003
	Alteration	31 (68.9)	
	Wild type	14 (31.1)	

*Indicates overall survival was significantly different between low-grade glioma (WHO grade II) and high-grade glioma (WHO grades III and IV).

#### Histone alteration status and correlation

Among the 56 cases, H3K27 alteration status was identified in 45 cases, consisting of 31 H3K27 alterations and 14 wild-type cases **Table 2**). Among the 45 cases with H3K27 alteration assessment results, a shorter overall survival was correlated with H3K27 alteration status (*P* = 0.003). Furthermore, H3K27 alteration was identified to be statistically correlated with the presence of stripes in the pontine (*P* = 0.035) and cerebellum involvement (*P* = 0.044). H3K27 alteration status showed no correlation with ADC, Cho/NAA, Cho/CR, or NAA/CR (**Table S1**). Pontine stripes in MR T2 were commonly observed in DIPGs (55%, 44/80 cases) and correlated with H3K27 alteration status (*P* = 0.006).

#### Treatment protocols and overall survival

**Figure 1:**
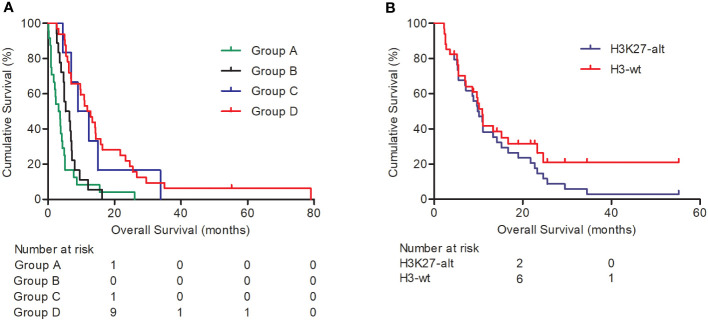
Kaplan–Meier survival analysis for DIPGs. Graph **(A)** shows the overall survival of DIPG patients in different treatment groups: Group A, clinical observation group; group B, stereotactic biopsy or open cranial surgery; group C, stereotactic biopsy plus radiotherapy and/or chemotherapy; group D, open cranial cytoreductive surgery plus radiotherapy and/or chemotherapy. Graph **(B)** indicates H3K27 alteration status predicts shorter overall survival (*P* = 0.004).

Among the 80 patients, 24 were clinically diagnosed as having DIPG according to MRI imaging and received no other clinical treatment. Another 18 patients only received stereotactic biopsy or open cranial surgery without subsequent radiotherapy and/or chemotherapy, another 6 patients received radiotherapy and/or chemotherapy after stereotactic biopsy, and the other 32 patients received open cranial tumor burden reduction surgery followed by radiotherapy and/or chemotherapy. Survival analysis indicated that patients receiving stereotactic biopsy or open cranial tumor burden reduction surgery plus radiotherapy and/or chemotherapy had a longer overall survival (*P* < 0.001, **Figure 1A**; **Table 3**), compared with the clinical observation group and the stereotactic biopsy/open cranial cytoreductive surgery group. The overall survival between the clinical observation group and the stereotactic biopsy or open cranial cytoreductive surgery group was of no statistical significance. The overall survival was significant between the other three groups (*P* < 0.001, **Table 3**).

**Table 3:** Treatment protocols correlated with OS of 80 DIPGs.

Parameters	Group A	Group B	Group C	Group D
*X* ± S	4.54 ± 5.69	6.15 ± 3.65	13.60 ± 10.58	16.71 ± 15.89
Group A	–	0.24	0.002	0.000*
Group B	0.24	–	0.000*	0.000*
Group C	0.002	0.000*	–	0.000*
Group D	0.000*	0.000*	0.000*	–

Group A, clinical observation; group B, stereotactic biopsy or open cranial surgery; group C, stereotactic biopsy plus radiotherapy and/or chemotherapy; group D, open cranial cytoreductive surgery plus radiotherapy and/or chemotherapy.

*Indicates P <0.001.

#### MRI features and overall survival

MRI imaging of the 80 cases was reviewed independently by two senior radiologists, and disagreement cases were reviewed again to reach an agreement. The detailed features of MRI images of the 80 cases are listed in **Table 4**. The medulla was involved in 58 cases, in which the pontomedullary sulcus was involved in 53 cases and the fundus of the fourth ventricle was involved in 33 cases. The midbrain was involved in 68 cases, in which the tectum was involved in 34 cases, the tegmentum was involved in 63 cases, and the cerebral peduncle was involved in 10 cases. The thalamus was involved in four cases, mainly involving the central-median part of the thalamus. The brachium was involved in 70 cases and the cerebellum was involved in 19 cases. Cerebellum involvement was identified to correlate with OS (*P* = 0.045, **Table 4**). No statistical significance was found between OS and other involved parts of the brainstem.

**Table 4:** Imaging parameters correlated with OS of 80 DIPGs.

Characteristics		No. (%) of patients	*P-*value
MRS parameters			
	Cho/Cr	–	0.037
	Cho/NAA	–	0.138
	NAA/Cr	–	0.840
ADC value			0.055
Enhancement			0.571
	Yes	52 (65.0)	
	No	28 (35.0)	
Necrosis			0.696
	Yes	25 (31.3)	
	No	55 (68.7)	
Intratumoral bleed			0.777
	Yes	7 (8.8)	
	No	73 (91.2)	
Cystic changes			0.793
	Yes	10 (12.5)	
	No	70 (87.5)	
Peritumoral edema			0.425
	Yes	25 (31.3)	
	No	55 (68.7)	
Hydrocephalus			0.681
	Yes	68 (85.0)	
	No	12 (15.0)	
Paraventricular edema			0.571
	Yes	62 (77.5)	
	No	18 (22.5)	
Pontine stripes			0.556
	Yes	44 (55.0)	
	No	36 (45.0)	

According to the shape and growth features of tumors in the pons, we divided DIPGs into two types: tropism growth and exophytic growth. The correlation between this classification and OS was of statistical significance (*P* = 0.023, **Table 5**), and tropism growth tumor had a much shorter overall survival.

**Table 5:** Tumor growth features in MRI imaging correlated with OS of 80 DIPGs.

Characteristics	No. (%) of patients	*P-*value
Midbrain involvement	68 (85.0)	0.320
Tectum	34 (50.0)	0.385
Tegmentum	63 (92.6)	0.816
Crus cerebri	10 (14.7)	0.381
Medulla involvement	59 (73.8)	0.205
Pontomedullary sulcus	53 (89.8)	0.079
Fourth ventricle fundus	33 (55.9)	0.433
Thalamus involvement	4 (5.0)	0.556
Brachium involvement	70 (87.5)	0.527
Cerebellum involvement	19 (23.8)	0.045
Classification based on growth pattern in MRI imaging		0.023
Without exophytic growth part	26 (32.5)	
With exophytic growth part	54 (67.5)	

**Figure 2:**
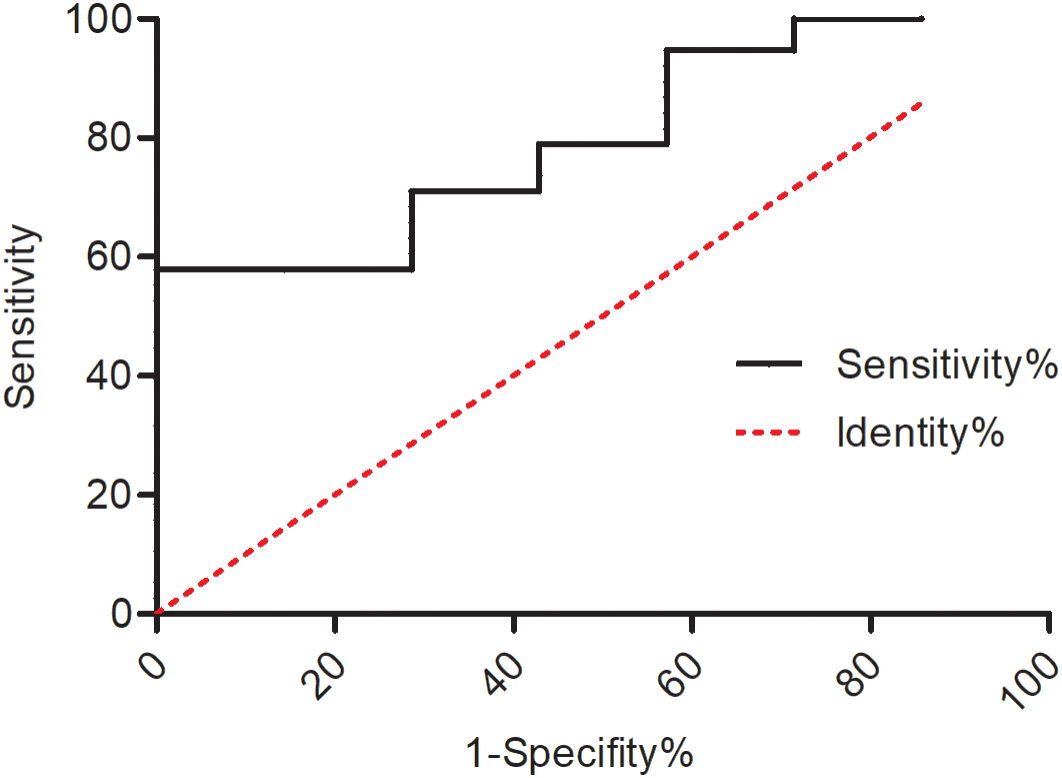
ROC curve of the predictive value of Cho/Cr for H3K27 alteration status (*P* = 0.012, cutoff value = 2.38, AUC = 0.801).

Imaging features that correlated with overall survival were calculated separately (**Table 5**). No statistical significance was found between OS and these MRI features. Quantitative analysis was performed between OS and the spectrum as well as diffuse restriction parameters. A lower rate of Cho/CR indicated a worse prognosis (*P* = 0.023, cutoff value = 1.66, AUC = 0.709, **Figure S1**). Furthermore, a lower rate of Cho/CR was also correlated with H3K27 alteration status (**Figure 2**) and a higher tumor grade (*P* = 0.002, **Table S2**). The ADC value, representing diffuse restriction feature, failed to identify any statistical correlation with OS.

With Cox analysis, H3K27 alteration indicated a worse prognosis and was an independent prognosis indicator for cases with H3K27 alteration test results (*P* = 0.004, **Figure 1B**).

## Discussion

### Correlation between clinical treatment and overall survival

**Figure 3:**
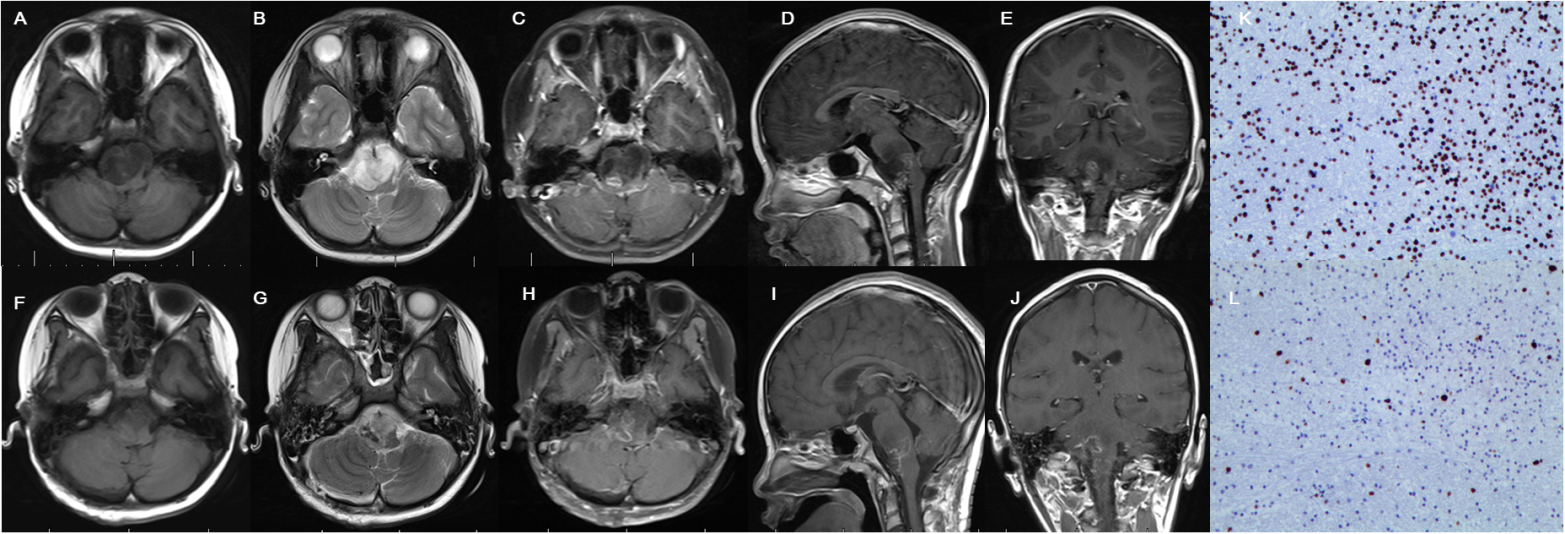
Cranial MRI examination revealed glioma located in the brainstem with extrapontine lesion, showing mixed hypointense and hyperintense in T1 **(A)** and hyperintense signal in T2 **(B)** and irregular enhancement **(C–E)** in preoperative images. Postoperative MRI confirmed partial resection **(F–J)**. Immunohistochemistry indicates H3K27M positive **(K)** and Ki-67 positive **(L)**.

Not all DIPGs were suitable for surgical removal. The exophytic portion of DIPGs could be safely resected with the use of intraoperative multimodal monitoring and neuronavigation techniques (6) (**Figure 3**). DIPGs with exophytic portions were more suitable for surgical resection and had a longer overall survival in this series.

Chemotherapy and radiotherapy have been recommended for DIPGs as treatment protocols for decades (7). However, resistance to chemotherapy and radiotherapy widely exists among DIPGs. Whether DIPG patients could benefit from chemotherapy and radiotherapy is still controversial. Within this series, through clinical observation, the overall survival was approximately 11 months, which was in accordance with previous reports (8). For DIPG patients who received chemotherapy and/or radiotherapy in this series, the overall survival was much longer than in the comparative groups (*P* < 0.001), which indicated that DIPG patients could benefit from chemotherapy and/or radiotherapy. Furthermore, we noticed from clinical practice that performing radiotherapy could induce the presence of enhancement and focal changes of DIPGs in MRI imaging, due to radiation-induced vascular changes within the tumor, which was noticed in previous case reports and needs further investigation (9).

The latest published study by our team indicates that the different locations of the brainstem tumor correlated with histone status and prognosis (3). This phenomenon did not exist in DIPGs in this series, which may be attributable to DIPGs mainly located in the pontine rather than in other parts of the brainstem.

### Correlation between MRI imaging and clinical features

With the development of stereotactic techniques and open cranial techniques, stereotactic biopsy or surgical biopsy is available for many DIPG patients under acceptable risks (10). However, due to the non-invasive advantages, DIPG is still mainly diagnosed based on MRI imaging and clinical manifestation. Furthermore, many features of the tumor can be identified through MRI images, for example, tumor growth pattern, enhancement, necrosis, hemorrhage, peritumoral edema, and hydrocephalus (11, 12). A survival prediction model as well as classification systems were also mentioned in previous reports (13, 14). These MRI features could provide rather important information for clinical diagnosis and therapy.

DIPG was defined as a tumor body mainly located in the pons. Within this study, we identified that DIPGs could also have different growth patterns with an extrapontine lesion on MRI imaging, which was also identified in a recently published article (15). In this series, at least 54 extrapontine lesions were observed in 80 cases (67.5%), which was similar to the report by Makepeace et al. (15). Among this series, more than 70% (58/80) of the DIPGs were found to involve the medulla, with the pontomedullary sulcus as the main affected part of the medulla, indicating that the tumor may involve the medulla mainly through the ventral part of the medulla. More than 80% (68/80) of the DIPGs in this series were found to involve the midbrain, with the tectum and tegmentum as the main affected parts of the midbrain, indicating that the tumor may involve the midbrain mainly through the dorsal part of the midbrain. These involved areas were the main locations where cranial nerves start over from the brainstem. In contrast to Makepeace’s report, we failed to identify a statistical correlation between overall survival and extrapontine lesion numbers or involvement of the middle cerebellar peduncles. We suggest that these findings may be valuable for risk stratification and radiation therapy planning in future clinical trials. Furthermore, these findings could be helpful in explaining why the lower cranial nerves as well as the long-tract defection signs were commonly identified as the early main clinical manifestations of DIPG patients.

### Correlation between MRI imaging and overall survival

Pontine stripes were distinct in the T2 series of many DIPGs, which have been mentioned previously (16) but not analyzed. This characteristic feature of DIPG was identified in more than 50% (44/80) of the cases in this study, indicating it to be a common MRI imaging feature in DIPG. We identified that the performance of pontine stripes was statistically correlated with H3K27 alteration (*P* = 0.006), although pontine stripes did not correlate with OS in this study.

Quantitative assessment of the spectrum and diffusion restriction has been used for the diagnosis of glioma and for the prediction of prognosis in supratentorial glioma studies (17–19). MRS was also used for differentiating brainstem glioma from non-tumoral diseases (20) and for indicating clinical progression (21). In this series, we identified that the lower rate of Cho/CR was correlated with a higher tumor grade and a shorter overall survival in DIPGs, with the tumor pathology grade an independent prognostic factor. These results indicate that a lower Cho/CR reflects malignant tumor growth features and could be used as a diagnostic indicator and a prognostic predictor.

Exophytic tumor growth has been recommended as a feature for brainstem tumor resectable indication in previous reports (22, 23). Exophytic tumor growth of DIPG accounts for 32.5% of the cases in this series (similar to 28% in previous reports (22)) and indicates a longer overall survival (*P* = 0.023). The reason for this may be because the exophytic growth pattern of the tumor causes less pressing or destruction of the brainstem, or because the exophytic tumor was more suitable for accepting neurosurgical resection, which provides tumor burden reduction and more tissue for pathological identification, allowing the generation of more effective, comprehensive therapy protocols. Other MRI imaging features, including enhancement, necrosis, cystic changes, and hemorrhage, did not correlate with the overall survival. Hydrocephalus and supratentorial periventricular edema were not correlated with overall survival.

## Conclusion

Some DIPGs were suitable for surgical removal for the purpose of diagnosis and cytoreductive surgery of the lesion. Postoperative comprehensive therapy is beneficial for the overall survival of the patients. H3K27 alteration status was correlated with overall survival. Cho/Cr was of predictive value for H3K27 alteration status; also, pontine stripes in the MR T2 series were commonly observed and were correlated with H3K27 alteration status, which needs further study with larger samples.

## Data availability statement

The raw data supporting the conclusions of this article will be made available by the authors, without undue reservation.

## Ethics statement

Written informed consent was obtained from the individual(s), and minor(s)’ legal guardian/next of kin, for the publication of any potentially identifiable images or data included in this article.

## Author contributions

PZ, YD, and GG carried out the clinical data collection, statistical analysis, and manuscript drafting and modification. DX and TX helped in the clinical data collection and statistical analysis. LQ and CP helped in the clinical data collection. YL and LZ conceived the study and participated in the design of the study and modified the manuscript. All authors contributed to the article and approved the submitted version.
