# Supplementary material for: Clinical, pathological, and radiological features of 80 pediatric diffuse intrinsic pontine gliomas: A single-institute study
Source: Front Oncol. 2023 Feb 7;13:1007393. doi: 10.3389/fonc.2023.1007393 (PMC9941347; doi:10.3389/fonc.2023.1007393)

Supplementary Tables

| Table s1.Imaging parameters correlated with H3K27M mutation status of 45 DIPGs | |
| --- | --- |
| Parameters | *P* Value |
| Age | 0.145 |
| Sex | 0.232 |
| Symptom duration time before diagnosis | 0.421 |
| Cho/Cr | 0.673 |
| Cho/NAA | 0.076 |
| NAA/Cr | 0.592 |
| ADC Value | 0.496 |
| Enhancement | 0.420 |
| Necrosis | 0.763 |
| Intratumoral Bleed | 0.479 |
| Cystic Changes | 0.404 |
| Peritumoral Edema | 0.371 |
| Hydrocephalus | 0.561 |
| Periventricular Edema | 0.392 |
| Pontine Stripes | 0.035 |
| Midbrain Involving | 0.164 |
| Medulla Involving | 0.629 |
| Thalamus Involving | 0.338 |
| Cerebellum Involving | 0.044 |
| Brachium Involving | 0.662 |

| Table s2.Imaging parameters correlated with tumor grade of 56 DIPGs* | |
| --- | --- |
| Parameters | *P* Value |
| Age | 0.881 |
| Sex | 0.829 |
| Symptom duration time before diagnosis | 0.407 |
| Cho/Cr | 0.002 |
| Cho/NAA | 0.436 |
| NAA/Cr | 0.215 |
| ADC Value | 0.085 |
| Enhancement | 0.058 |
| Necrosis | 0.024 |
| Intratumoral Bleed | 0.860 |
| Cystic Changes | 0.096 |
| Peritumoral Edema | 0.472 |
| Hydrocephalus | 0.898 |
| Periventricular Edema | 0.714 |
| Pontine Stripes | 0.982 |
| Midbrain Involving | 0.898 |
| Medulla Involving | 0.870 |
| Thalamus Involving | 0.931 |
| Cerebellum Involving | 0.733 |
| Brachium Involving | 0.931 |
| #Tumor were classified as low grade tumor(WHO Grade Ⅱ)and high grade tumor (WHO Grade Ⅲ and Ⅳ) | |
|

**Supplementary Figures**

Supplementary Figure s1. ROC curve of predictive value of Cho/Cr for long survival (＞12 months). (P=0.023,Cut-off Value=1.66,AUC=0.709)


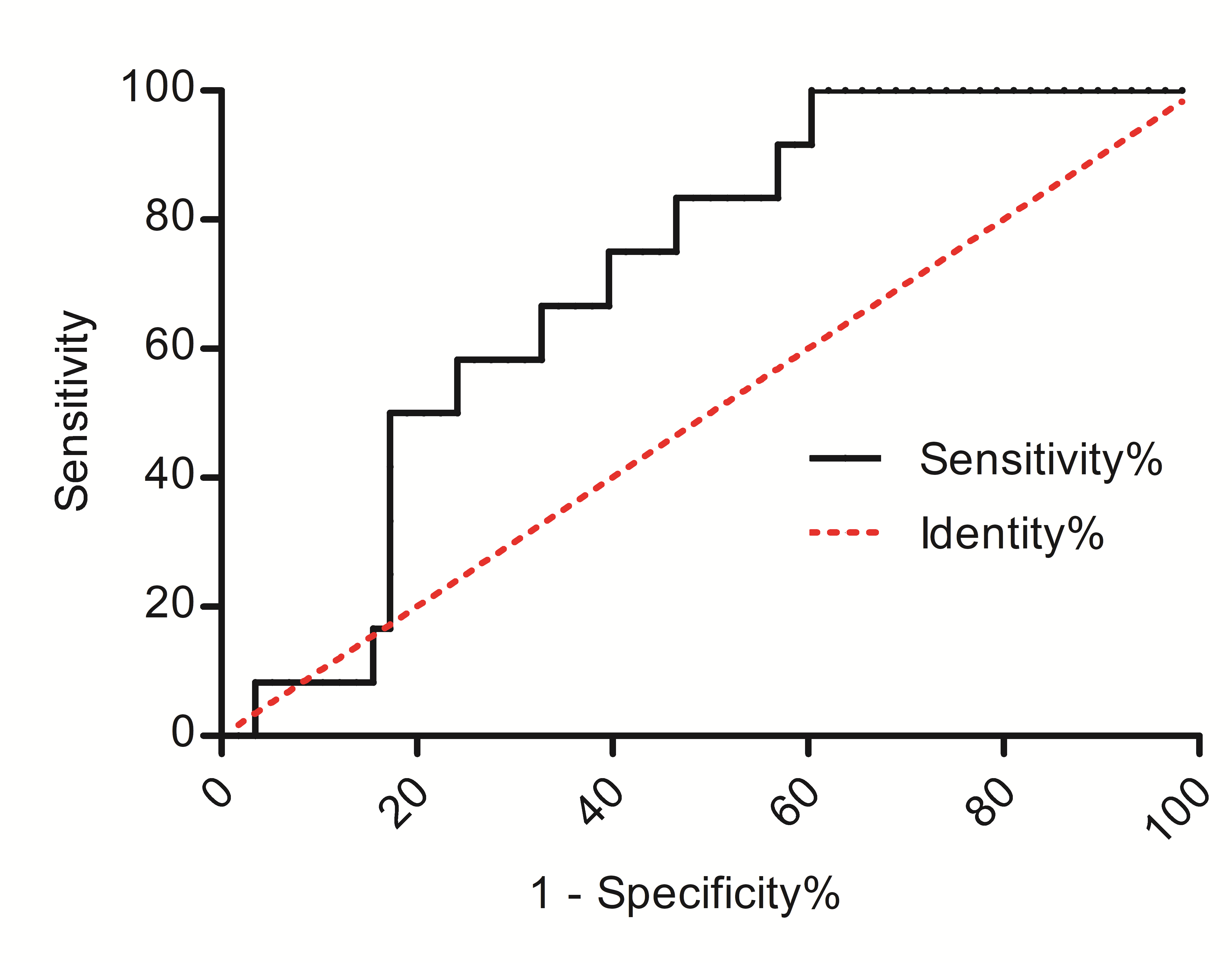

Supplement: Supplementary file 1 [file DataSheet_1.doc]
